# Duloxetine hydro­chloride

**DOI:** 10.1107/S1600536809033996

**Published:** 2009-08-29

**Authors:** Mohan Bhadbhade, James Hook, Chris Marjo, Anne Rich, Qinghong Lin

**Affiliations:** a UNSW Analytical Centre, The University of New South Wales, Sydney, NSW 2052, Australia; b Arrow Laboratories Ltd, Croydon South, Victoria 3136, Australia

## Abstract

The title compound [systematic name: *N*-methyl-3-(1-naphth­yloxy)-3-(2-thien­yl)propan-1-aminium chloride], C_18_H_20_NOS^+^·Cl^−^, was crystallized from 1,4-dioxane. Twofold rotational disorder exhibited by the thio­phene ring in a 0.580 (5):0.420 (5) ratio represents two different conformations of the mol­ecule that exist in the same crystal form. The crystal structure contains strong N—H⋯Cl hydrogen bonds.

## Related literature

For therapeutic properties of duloxetine hydro­chloride, see Waitekus & Kirkpatrick (2004 ▶). For related structures, see: Brenna *et al.* (2007 ▶); Tao *et al.* (2008 ▶). The title compound is reported to have different polymorphs on the basis of X-ray powder diffraction data, see: Ini *et al.* (2006 ▶).
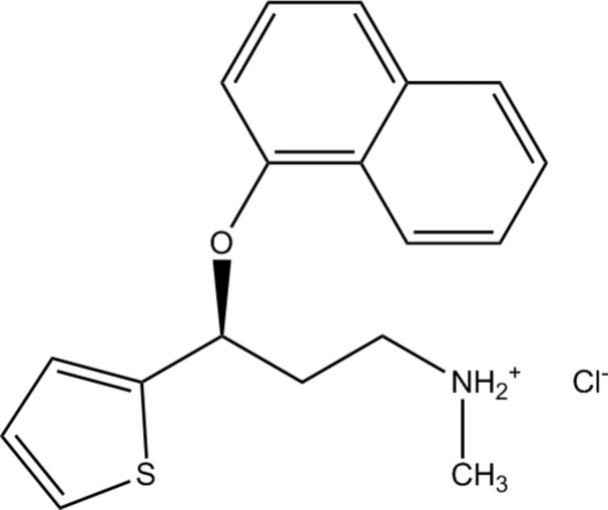


## Experimental

### 

#### Crystal data

C_18_H_20_NOS^+^·Cl^−^
*M* *_r_* = 333.86
Monoclinic, 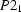

*a* = 9.7453 (10) Å
*b* = 6.9227 (7) Å
*c* = 13.4247 (16) Å
β = 109.432 (4)°
*V* = 854.09 (16) Å^3^
*Z* = 2
Mo *K*α radiation
μ = 0.35 mm^−1^
*T* = 150 K
0.38 × 0.08 × 0.03 mm

#### Data collection

Bruker Kappa APEXII CCD area-detector diffractometer
Absorption correction: multi-scan (*SADABS*; Sheldrick, 2003 ▶) *T*_min_ = 0.879, *T*_max_ = 0.990
6386 measured reflections
2947 independent reflections
2255 reflections with *I* > 2σ(*I*)
*R*_int_ = 0.058

#### Refinement

*R*[*F* ^2^ > 2σ(*F* ^2^)] = 0.044
*wR*(*F* ^2^) = 0.131
*S* = 0.79
2947 reflections
237 parameters
110 restraints
H-atom parameters constrained
Δρ_max_ = 0.17 e Å^−3^
Δρ_min_ = −0.21 e Å^−3^
Absolute structure: Flack (1983 ▶), 1309 Friedel pairs
Flack parameter: −0.05 (10)

### 

Data collection: *APEX2* (Bruker, 2007 ▶); cell refinement: *SAINT* (Bruker, 2007 ▶); data reduction: *SAINT*; program(s) used to solve structure: *SHELXS97* (Sheldrick, 2008 ▶); program(s) used to refine structure: *SHELXL97* (Sheldrick, 2008 ▶); molecular graphics: *SHELXTL-Plus* (Sheldrick, 2008 ▶); software used to prepare material for publication: *SHELXL97*.

## Supplementary Material

Crystal structure: contains datablocks I, global. DOI: 10.1107/S1600536809033996/bg2289sup1.cif

Structure factors: contains datablocks I. DOI: 10.1107/S1600536809033996/bg2289Isup2.hkl

Additional supplementary materials:  crystallographic information; 3D view; checkCIF report

## Figures and Tables

**Table 1:** Hydrogen-bond geometry (Å, °)

*D*—H⋯*A*	*D*—H	H⋯*A*	*D*⋯*A*	*D*—H⋯*A*
N1—H1*A*⋯Cl1^i^	0.92	2.23	3.113 (3)	161
N1—H1*B*⋯Cl1	0.92	2.18	3.087 (3)	170

Symmetry code: (i) 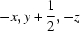.

**Fig. 1.**
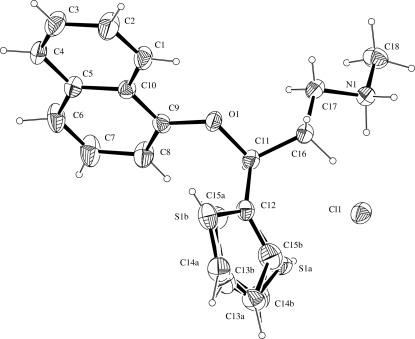
Molecular view of 1. Displacement ellipsoids drawn at 50% level.

**Fig. 2.**
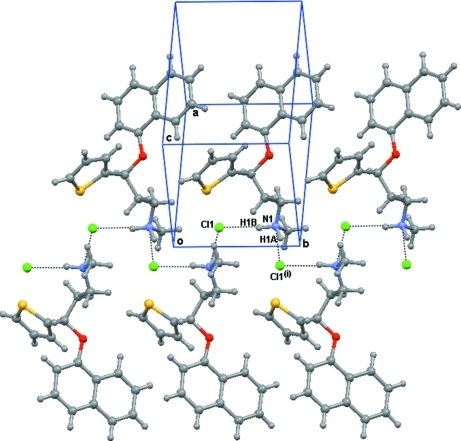
Packing view of1 showing N—H···Cl interactions.

## supplementary crystallographic information

### Comment

Duloxetine hydrochloride (1) is a new generation drug indicated for the management of major depressive disorders as well as for neuropathic pain (Waitekus, *et al.*, 2004). The compound 1 is reported to have different polymorphs on the basis of X-ray powder diffraction data (Ini *et al.*, 2006), but no single-crystal structure has as yet been presented. The only structures reported are that of a related racemic precursor (Tao, *et al.*, 2008) and of a regioisomer (Brenna *et al.*, 2007). Herein we report the structure of the drug itself (Fig. 1).

In the crystal structure, the thiophene ring is disordered over two positions obtained by 180 degree rotation about C11—C12 bond in a 0.580/0.420 (5) ratio. The same disorder with similar occupancies was also observed in the structure of an impurity (Brenna *et al.*, 2007). These two orientations represent two different molecular conformations that exist in the same crystal structure; in one of them (minor occupancy) the S atom makes a short intramolecular contact with the oxygen atom (S···O = 2.957 Å). The thiophene and naphthyl units are almost perpendicular to each other (angle between their mean planes 87.9 (1) °).

The crystal packing (Fig. 2) shows that both the H-atoms attached to the N atom of the side chain make strong almost linear H-bonding contacts with the chloride ion.

### Experimental

Microcrystalline powder of (1) was supplied by Arrow Laboratories Ltd.,Croydon, Australia. Recrystallization of this powder by slow evaporation was attempted in acetonitrile, 1,4-dioxane,chlorobenzene and 2-propanol. Suitable single crystals in the form of thin plates were grown from the first three solvents, crystals from chlorobenzene were very thin silky fibres unsuitable for single-crystal analysis. Crystals from the first three solvents yielded the same monoclinic P2(1) form having unit-cell parameters as given in Table 1. Amongst these, better quality crystals were obtained from 1,4-dioxane, which were used for further structural analysis.

### Refinement

The twofold disorder of the thiophene ring noted first in the E-map at the structure solution stage (two strong peaks and two long bonds instead of one), was confirmed subsequently in the full-matrix least-squares refinement. The molecular geometry for this ring was refined with restrained bond and angles. H atoms were idealized at their expected positions and allowed to ride both in coordinates (C—H = 0.96–0.99, N–H = 0.92 Å), as well as in their isotropic displacement factors (*U*ĩso~(H) = 1.2/1.5× U~equiv~(host).

### Crystal data

**Table:**

C_18_H_20_NOS^+^·Cl^−^	*F*(000) = 352
*M**_r_* = 333.86	*D*_x_ = 1.298 Mg m^−^^3^
Monoclinic, *P*2_1_	Mo *K*α radiation, λ = 0.71073 Å
Hall symbol: P 2yb	Cell parameters from 1635 reflections
*a* = 9.7453 (10) Å	θ = 2.3–22.1°
*b* = 6.9227 (7) Å	µ = 0.35 mm^−^^1^
*c* = 13.4247 (16) Å	*T* = 150 K
β = 109.432 (4)°	Thin Plates, colourless
*V* = 854.09 (16) Å^3^	0.38 × 0.08 × 0.03 mm
*Z* = 2	

### Data collection

**Table:**

Bruker Kappa APEXII CCD area-detector diffractometer	2947 independent reflections
Radiation source: fine-focus sealed tube	2255 reflections with *I* > 2σ(*I*)
graphite	*R*_int_ = 0.058
φ scans, and ω scans with κ offsets	θ_max_ = 25.0°, θ_min_ = 1.6°
Absorption correction: multi-scan (*SADABS*; Sheldrick, 2003)	*h* = −10→11
*T*_min_ = 0.879, *T*_max_ = 0.990	*k* = −8→8
6386 measured reflections	*l* = −15→15

### Refinement

**Table:**

Refinement on *F*^2^	Secondary atom site location: difference Fourier map
Least-squares matrix: full	Hydrogen site location: inferred from neighbouring sites
*R*[*F*^2^ > 2σ(*F*^2^)] = 0.044	H-atom parameters constrained
*wR*(*F*^2^) = 0.131	*w* = 1/[σ^2^(*F*_o_^2^) + (0.1*P*)^2^ + 0.0292*P*] where *P* = (*F*_o_^2^ + 2*F*_c_^2^)/3
*S* = 0.79	(Δ/σ)_max_ = 0.005
2947 reflections	Δρ_max_ = 0.17 e Å^−^^3^
237 parameters	Δρ_min_ = −0.21 e Å^−^^3^
110 restraints	Absolute structure: Flack (1983), 1309 Friedel pairs
Primary atom site location: structure-invariant direct methods	Flack parameter: −0.05 (10)

### Special details

**Table:**

Geometry. All e.s.d.'s (except the e.s.d. in the dihedral angle between two l.s. planes) are estimated using the full covariance matrix. The cell e.s.d.'s are taken into account individually in the estimation of e.s.d.'s in distances, angles and torsion angles; correlations between e.s.d.'s in cell parameters are only used when they are defined by crystal symmetry. An approximate (isotropic) treatment of cell e.s.d.'s is used for estimating e.s.d.'s involving l.s. planes.
Refinement. Refinement of *F*^2^ against ALL reflections. The weighted *R*-factor *wR* and goodness of fit *S* are based on *F*^2^, conventional *R*-factors *R* are based on *F*, with *F* set to zero for negative *F*^2^. The threshold expression of *F*^2^ > σ(*F*^2^) is used only for calculating *R*-factors(gt) *etc*. and is not relevant to the choice of reflections for refinement. *R*-factors based on *F*^2^ are statistically about twice as large as those based on *F*, and *R*- factors based on ALL data will be even larger.

### Fractional atomic coordinates and isotropic or equivalent isotropic displacement parameters (Å^2^)

**Table:**

	*x*	*y*	*z*	*U*_iso_*/*U*_eq_	Occ. (<1)
C1	0.7076 (5)	1.0388 (6)	0.3732 (3)	0.0414 (10)	
H1	0.6473	1.0546	0.3018	0.050*	
C2	0.8113 (5)	1.1721 (7)	0.4181 (3)	0.0559 (13)	
H2	0.8222	1.2804	0.3779	0.067*	
C3	0.9031 (5)	1.1525 (7)	0.5234 (3)	0.0498 (12)	
H3	0.9769	1.2456	0.5538	0.060*	
C4	0.8856 (4)	0.9990 (6)	0.5818 (3)	0.0404 (10)	
H4	0.9465	0.9877	0.6534	0.048*	
C5	0.7784 (4)	0.8557 (6)	0.5376 (2)	0.0333 (8)	
C6	0.7619 (5)	0.6944 (7)	0.5957 (3)	0.0488 (12)	
H6	0.8217	0.6804	0.6674	0.059*	
C7	0.6613 (5)	0.5596 (7)	0.5500 (3)	0.0594 (14)	
H7	0.6518	0.4508	0.5904	0.071*	
C8	0.5696 (5)	0.5748 (7)	0.4444 (3)	0.0453 (11)	
H8	0.5007	0.4764	0.4134	0.054*	
C9	0.5811 (4)	0.7331 (5)	0.3870 (3)	0.0302 (9)	
C10	0.6874 (4)	0.8761 (5)	0.4309 (3)	0.0285 (8)	
C11	0.3962 (4)	0.6244 (5)	0.2248 (3)	0.0287 (8)	
H11	0.3506	0.5543	0.2709	0.034*	
C16	0.2800 (4)	0.7340 (5)	0.1395 (3)	0.0334 (9)	
H16A	0.2233	0.6416	0.0851	0.040*	
H16B	0.3277	0.8263	0.1051	0.040*	
C17	0.1769 (4)	0.8439 (7)	0.1826 (3)	0.0395 (9)	
H17A	0.1988	0.8111	0.2581	0.047*	
H17B	0.1925	0.9844	0.1776	0.047*	
C18	−0.0810 (4)	0.8802 (6)	0.1698 (3)	0.0449 (10)	
H18A	−0.0634	0.8265	0.2405	0.067*	
H18B	−0.1802	0.8483	0.1246	0.067*	
H18C	−0.0694	1.0209	0.1746	0.067*	
Cl1	0.02776 (10)	0.35210 (15)	0.11567 (7)	0.0385 (3)	
N1	0.0237 (3)	0.7976 (4)	0.1242 (2)	0.0334 (7)	
H1A	0.0015	0.8416	0.0560	0.040*	
H1B	0.0131	0.6655	0.1214	0.040*	
O1	0.4920 (3)	0.7711 (3)	0.28494 (18)	0.0335 (6)	
C12	0.4783 (4)	0.4840 (6)	0.1806 (3)	0.0304 (9)	
S1A	0.4000 (4)	0.2836 (6)	0.1112 (4)	0.0390 (8)	0.580 (5)
C13A	0.5587 (15)	0.220 (3)	0.088 (2)	0.043 (2)	0.580 (5)
H13A	0.5703	0.1075	0.0508	0.051*	0.58
C14A	0.6634 (16)	0.353 (2)	0.1292 (18)	0.045 (2)	0.580 (5)
H14A	0.7575	0.3491	0.1227	0.054*	0.58
C15A	0.6158 (17)	0.500 (3)	0.184 (2)	0.042 (4)	0.580 (5)
H15A	0.6774	0.6027	0.2199	0.051*	0.58
S1B	0.6515 (6)	0.5208 (10)	0.1826 (7)	0.0410 (14)	0.420 (5)
C13B	0.656 (2)	0.310 (3)	0.119 (2)	0.042 (3)	0.420 (5)
H13B	0.7390	0.2624	0.1043	0.051*	0.42
C14B	0.526 (2)	0.221 (4)	0.091 (3)	0.045 (3)	0.420 (5)
H14B	0.5030	0.1062	0.0498	0.054*	0.42
C15B	0.427 (2)	0.318 (3)	0.131 (2)	0.053 (5)	0.420 (5)
H15B	0.3325	0.2691	0.1237	0.063*	0.42

### Atomic displacement parameters (Å^2^)

**Table:**

	*U*^11^	*U*^22^	*U*^33^	*U*^12^	*U*^13^	*U*^23^
C12	0.030 (2)	0.034 (2)	0.0272 (18)	−0.0013 (18)	0.0086 (16)	0.0036 (16)
S1A	0.0360 (14)	0.0354 (13)	0.0453 (18)	0.0021 (11)	0.0130 (14)	−0.0108 (10)
C13A	0.046 (6)	0.048 (5)	0.038 (5)	0.018 (5)	0.019 (6)	−0.002 (4)
C14A	0.039 (4)	0.060 (7)	0.042 (5)	0.009 (4)	0.021 (4)	−0.001 (5)
C15A	0.044 (9)	0.049 (6)	0.042 (5)	−0.005 (6)	0.025 (6)	−0.001 (4)
S1B	0.024 (2)	0.056 (2)	0.042 (2)	−0.0013 (18)	0.0099 (19)	0.0093 (17)
C13B	0.042 (5)	0.051 (6)	0.037 (7)	0.009 (5)	0.017 (5)	0.015 (5)
C14B	0.045 (7)	0.050 (6)	0.036 (6)	0.005 (6)	0.010 (6)	0.003 (5)
C15B	0.043 (7)	0.062 (12)	0.055 (11)	0.005 (7)	0.018 (8)	0.003 (8)
C1	0.047 (2)	0.038 (2)	0.0303 (19)	−0.007 (2)	0.0003 (18)	0.0082 (18)
C2	0.062 (3)	0.048 (3)	0.043 (2)	−0.020 (3)	−0.002 (2)	0.010 (2)
C3	0.051 (3)	0.045 (3)	0.043 (2)	−0.017 (2)	0.002 (2)	−0.003 (2)
C4	0.040 (2)	0.046 (2)	0.0269 (19)	−0.002 (2)	−0.0007 (17)	−0.0027 (19)
C5	0.034 (2)	0.0372 (19)	0.0269 (17)	−0.003 (2)	0.0081 (15)	−0.001 (2)
C6	0.046 (3)	0.058 (3)	0.032 (2)	−0.010 (2)	−0.0012 (19)	0.019 (2)
C7	0.060 (3)	0.061 (3)	0.042 (2)	−0.020 (3)	−0.004 (2)	0.028 (2)
C8	0.044 (2)	0.043 (2)	0.040 (2)	−0.014 (2)	0.0024 (19)	0.010 (2)
C9	0.032 (2)	0.032 (2)	0.0261 (18)	0.0051 (17)	0.0090 (15)	0.0010 (15)
C10	0.0295 (19)	0.031 (2)	0.0250 (16)	0.0001 (17)	0.0092 (14)	−0.0023 (16)
C11	0.026 (2)	0.031 (2)	0.0268 (18)	−0.0037 (15)	0.0066 (16)	0.0011 (14)
C16	0.029 (2)	0.035 (2)	0.0308 (19)	0.0003 (18)	0.0029 (16)	−0.0060 (16)
C17	0.033 (2)	0.040 (2)	0.0382 (19)	0.002 (2)	0.0021 (16)	−0.015 (2)
C18	0.039 (2)	0.040 (2)	0.060 (2)	0.003 (2)	0.023 (2)	0.000 (2)
Cl1	0.0438 (6)	0.0345 (5)	0.0345 (5)	−0.0070 (5)	0.0095 (4)	0.0009 (4)
N1	0.0348 (17)	0.0295 (17)	0.0335 (16)	−0.0006 (14)	0.0080 (14)	0.0003 (13)
O1	0.0365 (14)	0.0308 (13)	0.0254 (12)	−0.0054 (12)	−0.0003 (11)	−0.0003 (10)

### Geometric parameters (Å, °)

**Table:**

C1—C2	1.353 (6)	C17—H17A	0.9900
C1—C10	1.418 (5)	C17—H17B	0.9900
C1—H1	0.9500	C18—N1	1.469 (5)
C2—C3	1.406 (5)	C18—H18A	0.9800
C2—H2	0.9500	C18—H18B	0.9800
C3—C4	1.364 (6)	C18—H18C	0.9800
C3—H3	0.9500	N1—H1A	0.9200
C4—C5	1.420 (6)	N1—H1B	0.9200
C4—H4	0.9500	C12—C15A	1.332 (14)
C5—C6	1.402 (6)	C12—C15B	1.341 (16)
C5—C10	1.418 (4)	C12—C11	1.501 (5)
C6—C7	1.344 (6)	C12—S1B	1.699 (7)
C6—H6	0.9500	C12—S1A	1.703 (5)
C7—C8	1.406 (5)	S1A—C13A	1.734 (10)
C7—H7	0.9500	C13A—C14A	1.351 (10)
C8—C9	1.365 (5)	C13A—H13A	0.9500
C8—H8	0.9500	C14A—C15A	1.420 (14)
C9—O1	1.381 (4)	C14A—H14A	0.9500
C9—C10	1.413 (5)	C15A—H15A	0.9500
C11—O1	1.433 (4)	S1B—C13B	1.699 (14)
C11—C16	1.519 (5)	C13B—C14B	1.351 (11)
C11—H11	1.0000	C13B—H13B	0.9500
C16—C17	1.520 (5)	C14B—C15B	1.421 (15)
C16—H16A	0.9900	C14B—H14B	0.9500
C16—H16B	0.9900	C15B—H15B	0.9500
C17—N1	1.472 (4)		
			
C2—C1—C10	121.2 (3)	N1—C17—H17B	109.3
C2—C1—H1	119.4	C16—C17—H17B	109.3
C10—C1—H1	119.4	H17A—C17—H17B	107.9
C1—C2—C3	120.9 (4)	N1—C18—H18A	109.5
C1—C2—H2	119.5	N1—C18—H18B	109.5
C3—C2—H2	119.5	H18A—C18—H18B	109.5
C4—C3—C2	119.5 (4)	N1—C18—H18C	109.5
C4—C3—H3	120.2	H18A—C18—H18C	109.5
C2—C3—H3	120.2	H18B—C18—H18C	109.5
C3—C4—C5	121.3 (3)	C18—N1—C17	114.6 (3)
C3—C4—H4	119.3	C18—N1—H1A	108.6
C5—C4—H4	119.3	C17—N1—H1A	108.6
C6—C5—C10	119.5 (4)	C18—N1—H1B	108.6
C6—C5—C4	121.9 (3)	C17—N1—H1B	108.6
C10—C5—C4	118.6 (3)	H1A—N1—H1B	107.6
C7—C6—C5	120.0 (4)	C9—O1—C11	120.0 (3)
C7—C6—H6	120.0	C15A—C12—C15B	107.3 (10)
C5—C6—H6	120.0	C15A—C12—C11	126.5 (7)
C6—C7—C8	122.0 (4)	C15B—C12—C11	126.2 (8)
C6—C7—H7	119.0	C15B—C12—S1B	110.1 (8)
C8—C7—H7	119.0	C11—C12—S1B	123.7 (3)
C9—C8—C7	119.0 (4)	C15A—C12—S1A	110.5 (7)
C9—C8—H8	120.5	C11—C12—S1A	122.9 (3)
C7—C8—H8	120.5	S1B—C12—S1A	113.1 (3)
C8—C9—O1	124.8 (3)	C12—S1A—C13A	92.4 (4)
C8—C9—C10	120.9 (3)	C14A—C13A—S1A	110.4 (8)
O1—C9—C10	114.4 (3)	C14A—C13A—H13A	124.8
C9—C10—C1	123.0 (3)	S1A—C13A—H13A	124.8
C9—C10—C5	118.5 (3)	C13A—C14A—C15A	111.8 (10)
C1—C10—C5	118.5 (3)	C13A—C14A—H14A	124.1
O1—C11—C12	110.4 (3)	C15A—C14A—H14A	124.1
O1—C11—C16	104.7 (3)	C12—C15A—C14A	114.8 (11)
C12—C11—C16	112.8 (3)	C12—C15A—H15A	122.6
O1—C11—H11	109.6	C14A—C15A—H15A	122.6
C12—C11—H11	109.6	C13B—S1B—C12	93.3 (7)
C16—C11—H11	109.6	C14B—C13B—S1B	110.6 (11)
C11—C16—C17	112.6 (3)	C14B—C13B—H13B	124.7
C11—C16—H16A	109.1	S1B—C13B—H13B	124.7
C17—C16—H16A	109.1	C13B—C14B—C15B	112.1 (12)
C11—C16—H16B	109.1	C13B—C14B—H14B	124.0
C17—C16—H16B	109.1	C15B—C14B—H14B	124.0
H16A—C16—H16B	107.8	C12—C15B—C14B	113.7 (12)
N1—C17—C16	111.7 (3)	C12—C15B—H15B	123.2
N1—C17—H17A	109.3	C14B—C15B—H15B	123.2
C16—C17—H17A	109.3		

### Hydrogen-bond geometry (Å, °)

**Table:**

*D*—H···*A*	*D*—H	H···*A*	*D*···*A*	*D*—H···*A*
N1—H1A···Cl1^i^	0.92	2.23	3.113 (3)	161
N1—H1B···Cl1	0.92	2.18	3.087 (3)	170

Symmetry codes: (i) −*x*, *y*+1/2, −*z*.
